# Gut microbiota composition and dysbiosis signatures associated with complex gastrointestinal and urological conditions in neonates: a case series study

**DOI:** 10.3389/fmed.2026.1917675

**Published:** 2026-10-23

**Authors:** Marsiglia Riccardo, Domenico Umberto De Rose, Pane Stefania, Chiara Marangelo, Guglielmo Salvatori, Fabio Fusaro, Andrea Conforti, Irma Capolupo, Lorenza Putignani, Andrea Dotta

**Affiliations:** 1 Unit of Microbiome, Bambino Gesù Children’s Hospital, IRCCS, Rome, Italy; 2 Neonatal Intensive Care Unit, Bambino Gesù Children’s Hospital, IRCCS, Rome, Italy; 3 Unit of Microbiomics, Bambino Gesù Children’s Hospital, IRCCS, Rome, Italy; 4 Neonatal and Pediatric Surgery Unit, Bambino Gesù Children’s Hospital, IRCCS, Rome, Italy; 5 Department of Life Science, Health, and Health Professions, Link Campus University, Rome, Italy

**Keywords:** dysbiosis, gastrointestinal surgery, gut microbiota, newborns, urological conditions

## Abstract

**Introduction:**

During the first year of life, the gut microbiota (GM) is influenced by several factors, including delivery mode, feeding practices (breast milk vs. formula milk), antibiotic therapy, and preterm birth. Surgical interventions and complex congenital conditions may further alter microbial colonization, potentially contributing to complications and systemic inflammation. This study aimed to investigate alterations in the GM of newborns with complex surgical, urological, and gastrointestinal conditions, including Hirschsprung's disease.

**Methods:**

GM composition was analyzed in five newborns with complex clinical conditions to investigate potential associations between microbial alterations and underlying diseases.

**Results:**

A consistent reduction in beneficial commensal bacteria, particularly Bifidobacterium and Lactobacillus spp., was observed, alongside an increased abundance of potential opportunistic pathogens, mainly belonging to the phylum Proteobacteria. These findings revealed common signatures of dysbiosis, as well as potential disease-specific microbial patterns.

**Discussion:**

The findings suggest that early-life factors, including antibiotic exposure, prematurity, complex clinical conditions, and surgical interventions, may substantially affect microbial colonization. These alterations may be associated with an increased risk of complications and systemic inflammation. Further studies involving larger cohorts are needed to clarify the relationships between GM alterations and clinical outcomes in these vulnerable newborns.

## Introduction

In the first days of life of healthy term newborns, the most abundant colonizers of the intestinal microbiota are staphylococci, proteobacteria, and bifidobacteria (1). Then, over the ﬁrst few years of life of infants, the intestinal tract progresses from sterility to colonization with a variety of microbes (aerobic and anaerobic) to progressively become very similar to the adult intestine (2, 3). In particular, during the first year of life, the gut microbiota (GM) is relatively unstable (4) and is influenced by different factors including delivery mode and infant food (i.e., breast vs. formula milk) (5). The first colonizing species in the intestine of a term infant born spontaneously are a combination similar to the maternal vaginal microbiota (6), particularly *Lactobacillus* and *Prevotella* spp.; facultative anaerobes such as *Escherichia* spp.; and members of the Enterobacteriaceae family (7). In contrast, intestinal colonization in term infants born via a cesarean section is dominated by species from the maternal skin and the surrounding environment such as *Staphylococcus*, *Corynebacterium*, and *Propionibacterium* (8). The GM of newborns is also highly influenced by feeding (9–11). Indeed, exclusively breastfed infants show a uniform population of microbes in the gut microbiome with a high prevalence of Bifidobacteria, which are beneficial for intestinal health (12), followed by *Bacteroides* and *Clostridium* spp. (13).

However, GM of an infant is affected by various factors, including formula feeding, multiple courses of antibiotic therapy, and preterm birth (14, 15). Feeding formula milk with a higher amount of protein establishes a proteolytic metabolism. Furthermore, compared with the GM of breast milk–fed infants, the GM of formula-fed infants generally shows a decreased bacterial population, an increased diversity of bacterial species, and less prevalence of bifidobacteria (15), with a predominance of staphylococci, *Bacteroides* spp., clostridia, enterococci, enterobacteria, and *Atopobium* spp. (16, 17). In addition, prolonged antibiotic therapy in newborns may disrupt intestinal homeostasis and promote the onset of antibiotic-resistant pathogens, consequently increasing the risks of necrotizing enterocolitis (NEC), late-onset sepsis, and death (18). The GM of preterm infants is commonly characterized by the increased prevalence of proteobacteria (especially *Enterobacter* spp.) and coagulase-negative staphylococci (CoNS) species and by the lower prevalence of lactobacilli and bifidobacteria (14, 19–21), a condition that can lead to the development of NEC (22).

After a major medical insult, such as surgery, the GM undergoes a significant ecological collapse and a strong shift in the prevalence and function of intestinal microbes (23). In particular, the GM in neonates requiring surgery is affected by many factors, such as surgical stress, inflammation, intravenous nutrition, and antibiotic treatment (24). Hence, the GM composition undergoes a series of alterations, which are beneficial for the modulation of gut dysbiosis (15, 25). In this study, alterations in the GM of five newborns were examined in relation to complex surgical, urological, and gastrointestinal conditions, including Hirschsprung's disease, and clinical factors potentially influencing the microbial composition, such as antibiotic exposure, premature birth, and feeding, with the aim of exploring disease-specific microbial patterns and exogenous factors affecting them.

## Material and methods

### Patient enrollment and sample collection

The cases of Five patients are presented in this case series, and they were admitted to Bambino Gesù Children's Hospital (OPBG) in Rome, Italy. All procedures in this study were performed in accordance with the ethical standards of the institutional and national research committees and with the 1964 Declaration of Helsinki and its later amendments or comparable ethical standards. All parents were fully informed about the investigational nature of this study, and they signed the written informed consent forms for the use of the obtained clinical data. Personal data were restricted to essential information and were treated in a way that guarantees respect and privacy of the involved patients, as stated by the Italian Law (Legislative Decree No. 196 of 2003) about personal data protection. The study was approved by the Bambino Gesù Children’s Hospital ethics committee (approval no. 1196_OPBG_2016).

Stools were collected postsurgery from four of five patients. Stoma samples from the upper and lower stomach were also collected for case 1, while only the ileostomy sample was collected for case 2. After the collection of samples, they were transported under temperature-controlled conditions (4 °C) to the Microbiome Unit of the Bambino Gesù Children's Hospital and were stored at −80 °C until further microbiomics procedures. Clinical characteristics and sampling information of patients are reported in Table 1. For gut microbiota comparisons, 30 healthy controls (CTRLs) were selected from the reference Microbiome Special Collection of Bambino Gesù Children’s Hospital, Institute for Maternal and Child Health Burlo Garofolo (IRCCS), under the Biobanking and Biomolecular Resources Research Infrastructure (BBMRI) Biobank, which was established through an epidemiological survey conducted at the Microbiome Research Unit of OPBG (EUBIOM Project), a node of the BBMRI Infrastructure. The biobank comprises biological samples collected from healthy pediatric subjects, and the use of this sample collection was approved by the Ethics Committee of Bambino Gesù Children’s Hospital [protocol no. 2839_OPBG_2022; approval no. 1076 (26 July 2022); CE version 1.0, 05/09/2022 EUBIOM/2839_OPBG_2022]. CTRLs constituted an independent reference group. The patients were compared with 30 healthy CTRLs (full-term, no history of gastrointestinal diseases, non-hospitalized, and not undergoing antibiotic therapy). The CTRLs were age-matched with the patients and were specifically divided into two cohorts: 15 controls in the age range of 0–1 month and 15 controls in the age range of 2–12 months).

**Table 1:** Patient summary table: characterization of patients based on gestational weeks, birth weight, sex, delivery mode, diagnosis, surgical procedure, sample type, age at sampling time, infectious episodes, antibiotic exposure, feeding type, parental nutrition, probiotic exposure, and duration of hospitalization.

Patient characterization	Patient 1	Patient 2	Patient 3	Patient 4	Patient 5
Gestational weeks	34 GW	24 + 6 GW	38 GW	39 GW	38 GW
Birth weight (g)	2,160	870	2,750	3,430	3,280
Sex	Male	Male	Male	Female	Male
Delivery mode	Cesarean section	Cesarean section	Cesarean section	Operative vaginal delivery (vacuum extraction)	Cesarean section
Diagnosis	Congenital laryngeal cleft (type IV)	PDA, carrier of ileostomy	Trisomy 21, aganglionic megacolon, and intestinal obstruction	Megacolon and Hirschsprung’s disease	PUVs and bilateral vesicoureteral reflux, classified as grade V
Surgical procedure	Gastric bipartition and esophagus surgical recanalization (11 days of age)	Ileocolostomy (89 days of age)	Not required	Not required	Not required
Sample type	Stoma samples	Ileostomy sample	Stool sample	Stool sample	Stool sample
Age at the time of collection of stool/stoma and LPS samples	T1 (2 months) T2 (3 months)	3 months	7 months	10 days	5 months
Infectious episodes	*Escherichia coli*, *Pseudomonas aeruginosa*, *Staphylococcus aureus*, and *Stenotrophomonas maltophilia*	*Klebsiella pneumoniae*	No infectious complications occurred during the admission.	No infectious complications occurred during the admission.	*S. aureus* and *K. pneumoniae*
Antibiotic exposure	Meropenem, vancomycin, amikacin, oxacillin, gentamicin, piperacillin/tazobactam, metronidazole, and ceftolozane/tazobactam	Meropenem and intravenous immunoglobulins, vancomycin, and amikacin	Cefoxitin and metronidazole	Ampicillin, netilmicin, and metronidazole	Teicoplanin and meropenem.
Feeding type	Enteral nutrition (via gastrostomy)	Enteral nutrition	Enteral nutrition	Enteral nutrition	Enteral nutrition
Parental nutrition	Yes	Yes	Yes	Yes	No
Probiotic exposure	No	No	No	No	No
Duration of hospitalization (days)	168	47	125	17	20

GW, gestational weeks; PDA, large patent ductus arteriosus; PUVs, posterior urethral valves.

### Metataxonomy analysis: DNA extraction and next-generation sequencing pipelines

Briefly, DNA was extracted using a QIAamp Fast DNA Stool Mini Kit (Qiagen, Hilden, Germany) and the V3–V4 hypervariable region of the 16S rRNA was amplified using 2× KAPA HiFi HotStart ReadyMix (KAPA Biosystems Inc., Wilmington, MA, USA), according to the manufacturer's instructions. Illumina Nextera adapter primers were used to index the samples, after which they were sequenced on an Illumina MiSeq Platform (Illumina, San Diego, CA, USA).

For each sample, the V3–V4 region of the 16S rRNA gene was amplified via polymerase chain reaction (PCR) to obtain bacterial amplicon libraries (630 bp) using primers reported in the MiSeq rRNA Amplicon Sequencing protocol (Illumina, San Diego, CA, USA) (26). Internal PCR contaminations were excluded by using blank controls (no template). Sequencing was performed on an Illumina MiSeq platform (Illumina, San Diego, CA, USA). To avoid the batch effect and laboratory contamination, the protocol was standardized in terms of standardized operative procedures (SOP) with reference to reagent and of reproducibility of manual procedures according to staff training provided by the hospital. FASTQ files were processed using QIIME 2 (version 2024.5) (27). Sequence quality control, read denoising, chimera removal, paired-end read merging, and amplicon sequence variant (ASV) inference were performed using the DADA2 plugin (28). Taxonomic assignment was performed using the Greengenes2 plugin against the Greengenes2 reference database (http://www.greengenes.secondgenome.com, release 2022.10) (29). The ASV abundance table and taxonomic assignments were subsequently imported into R (version 4.3.2) for downstream analyses.

Furthermore, in addition to single sample analysis, we assigned a degree of dysbiosis (microbial dysbiosis index [MDI]) in patients by comparison with the GM profiles from CTRLs matched for age and gender, using three fecal samples (collected in three different days), based on a metagenomic method developed by our algorithm (OPBG patent no. EP3472611, granted on 15 May 2024; https://worldwide.espacenet.com/publicationDetails/biblio?CC = EP&NR = 3472611B1&KC = B1&FT = D&ND = 4&date=20240515&DB=&locale=). According to the patent, metagenomics was used to qualitatively and quantitatively characterize the GM profiles at the phylum, family, and genus taxon levels. The percentage quadratic dissimilarity index (Z) is given by Z = (½ × Σ (f_case_−f_controls_)2)1/2 × 100 (30), where f_case_ and f_controls_ are the median values of the taxa distribution at the phylum, family, and genus taxon levels of the GM of patients and CTRLs, respectively. The degree of dysbiosis was classified as mild (<25%), moderate (25%–35%), and high (>35%) according to an empirical algorithm developed during outpatient visit evaluation (31). In this study, MDI was exclusively used as an exploratory measure and not as a diagnostic parameter.

### Bioassays for lipopolysaccharide determinations

Accordingly, based on clinician request, two of five patients were assayed for serum lipopolysaccharides (LPS) to study inflammation triggered by LPS from Gram-negative bacteria. An enzyme-linked immunosorbent assay (ELISA) kit (Hycult Biotechnology, Uden, Netherlands) was used to measure LPS levels. Absorbance was measured in a microplate reader at 405 nm. Serum samples were analyzed undiluted and at 1:5, 1:10, and 1:20 dilutions according to the manufacturer’s instructions. LPS concentrations were calculated from the standard curve and corrected for the corresponding dilution factor. The 0.01–1.0 EU/mL range represents the reported physiological/reference range for serum LPS.

## Results

### Case 1

#### Clinical presentation

A preterm male infant—born at 34 weeks of gestational age (GA) via an urgent cesarean section due to polyhydramnios, premature rupture of membranes, and maternal liver enzyme elevation, with a birthweight of 2160 g—was transferred to our tertiary care center during his first week of life. Upon arrival, the patient required intubation and mechanical ventilation due to a congenital laryngeal cleft (type IV). A gastric bipartition surgery was performed to address feeding issues and airway protection. The postoperative course was complicated by gastric perforation with pneumoperitoneum, which required emergent surgical repair and placement of a new gastrostomy tube.

In the following days, the patient developed a tension pneumothorax, necessitating urgent chest tube placement. Tracheoesophageal reconstruction according to the Grillo technique was subsequently performed. Postoperatively, the infant remained pharmacologically paralyzed for 1 week, followed by another week of deep sedation for wound healing.

Later, airway evaluation via laryngotracheobronchoscopy revealed anterior subglottic stenosis, which required tracheostomy. During the same procedure, further endoscopic findings revealed significant adhesive fusion of the upper esophageal inlet. Radiographic contrast studies confirmed a severe proximal esophageal stricture.

Before further surgical correction could be undertaken and after a multidisciplinary discussion, the infant experienced clinical deterioration marked by respiratory distress, abdominal distension, and increased inflammatory marker levels. Abdominal ultrasound results revealed thickened bowel loops with intramural gas, raising concern for possible intestinal compromise. Broad-spectrum empiric antimicrobial therapy was initiated, which includes meropenem, amikacin, teicoplanin (after vancomycin discontinuation due to rash), and fluconazole. After this episode, enteral nutrition was cautiously started with hydrolyzed formula and progressively increased, reaching full enteral feeding at approximately 2 months of age. Blood and urine culture results remained negative, while cultures from the bronchoalveolar lavage (BAL) yielded *Stenotrophomonas maltophilia* at high colony counts, likely interpreted as likely colonization.

Subsequent endoscopic and radiological evaluation of the esophagus allowed for surgical recanalization. A 10 Fr nasogastric tube was positioned and anchored to the superior gastric pouch via the gastrostomy site. In the immediate postoperative period, the patient required supplemental high-flow oxygen and frequent airway suctioning due to increased secretions. A regimen of systemic and inhaled corticosteroids was initiated and managed for 72 h with notable clinical improvement. The patient was then transferred to the surgical ward in stable condition. During the hospital stay, ophthalmologic and audiologic screenings were within normal limits. Renal ultrasound results showed left-sided pyelectasis. Genetic testing and neuroimaging (cranial ultrasound and MRI) revealed no abnormalities.

At multidisciplinary follow-up, the infant underwent esophageal dilation and replacement of the gastrostomy tube with a low-profile 14 Fr device. Prior to discharge, tracheostomy care was optimized and immunizations were administered according to current guidelines. At discharge, the infant was in good general condition, tolerating full enteral feeds. Pulmonary auscultation revealed good bilateral air entry. The tracheostomy was patent and functioning, and the gastrostomy site was clean, with only minimal peristomal secretions.

During the hospital stay, the patient received meropenem, vancomycin, amikacin, oxacillin, gentamicin, piperacillin/tazobactam, metronidazole, and ceftolozane/tazobactam for repeated infectious episodes (*Escherichia coli*, *Pseudomonas aeruginosa*, *Staphylococcus aureus*, and *S. maltophilia*).

#### Gut microbiota

The GM of the patient was analyzed on both stool samples and gastrostomy contents (upper and lower stomach contents) at 1 month and 2 months after surgery (T_1_ and T_2_, respectively).

In particular, the GM maps at T_1_ with several features are shown in Figure 1.

**Figure 1:**
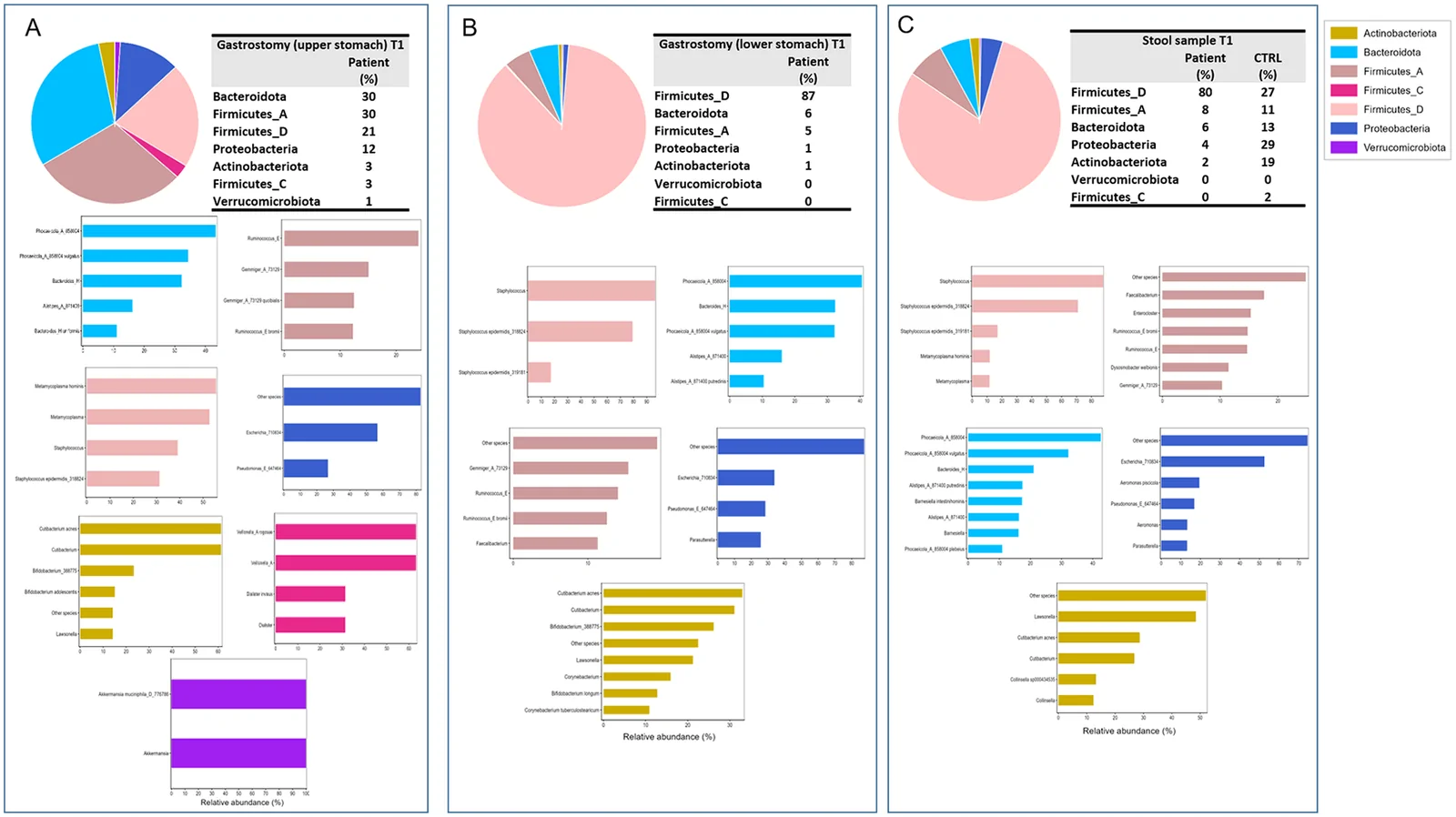
The GM profile of “case 1” on the gastrostomy content of upper **(A)** and lower **(B)** stomach and stool **(C)** samples 1 month after surgery (T_1_). “Other genus” and “other species” refer to ASVs greater than 1% of distribution but undefined.

The gastric contents of upper (Figure 1A) and lower (Figure 1B) stomach portions had a high prevalence of Firmicutes [especially, Firmicutes_A (i.e., *Staphylococcus*) and Firmicutes_D (*Ruminococcus_E*)], Bacteroidota (*Phocaeicola_A_858004*), and Proteobacteria (*Escherichia_710834*, *Pseudomonas_E_647464*). In particular, the lower stomach map showed a higher prevalence of Firmicutes (87% being Firmicutes_D) and a lower prevalence of Bacteroidota (6%) and Actinobacteriota (1%) than the upper stomach map.

GM analysis of stools (Figure 1C) showed a higher prevalence of Firmicutes (80% of Firmicutes_D and 8% of Firmicutes_A) in the patient than in CTRLs. The profile was also characterized by a decrease in the prevalence of Proteobacteria (4%), Bacteroidota (6%), and Actinobacteriota (2%). Later, stools and only the upper gastrostomy content were analyzed 2 months after surgery (T_2_).

The fecal GM profile at T_2_ showed a decrease in the prevalence of Firmicutes (34.6% of Firmicutes_D) and an increase in the prevalence of Bacteroidota (13.2%), Proteobacteria (17.2%) and Actinobacteriota (6.3%) compared with that at T_1_ (Figures 2a,b).

**Figure 2:**
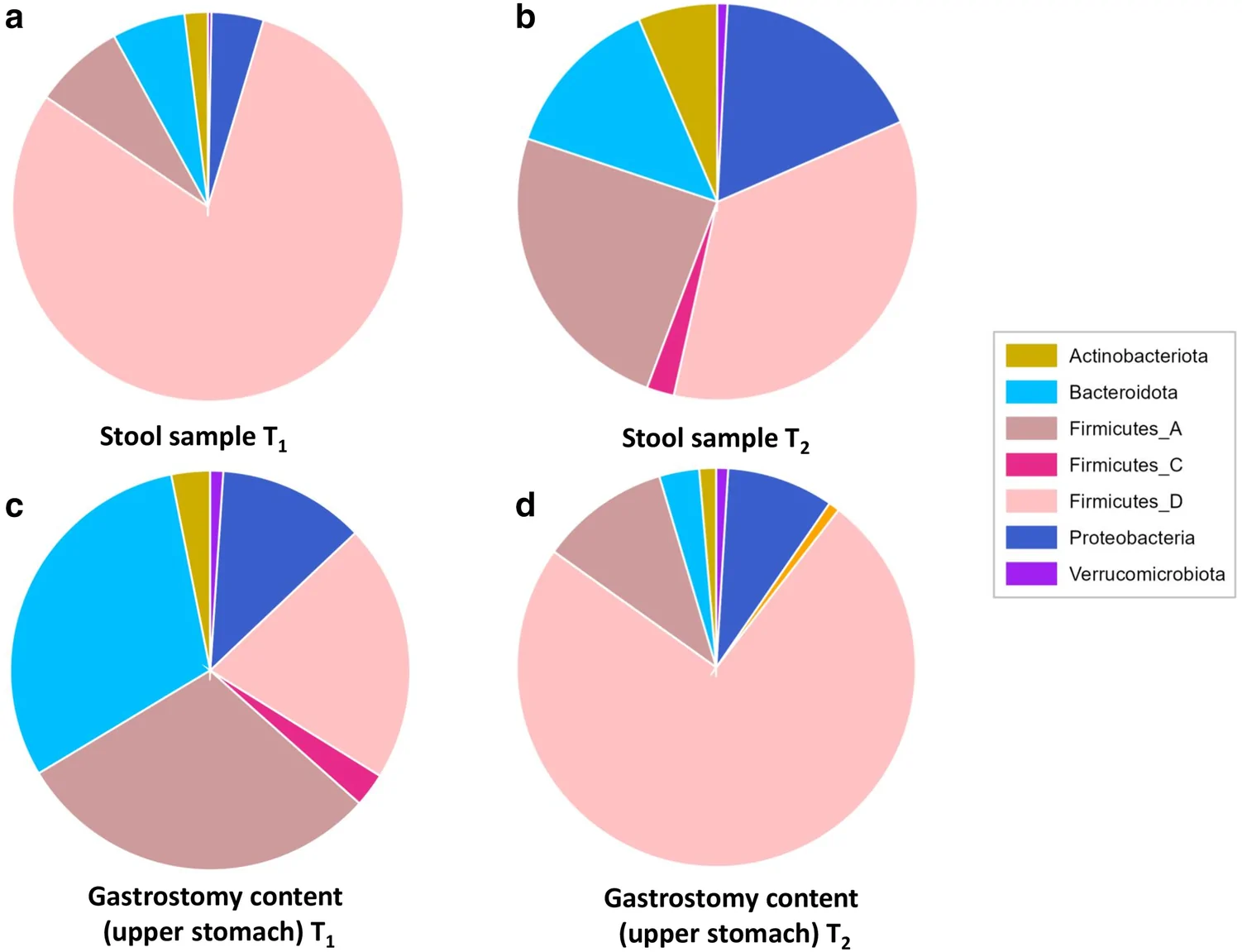
The GM profile of “case 1” on stools at T_1_ (1 month) **(a)** and T_2_ (2 months) **(b)** and on gastrostomy contents (upper stomach) at T_1_ (1 month) **(c)** and T_2_ (2 months) **(d)** after surgery.

In particular, compared with the GM profile of CTRLs, the GM profile of the patient at T_1_ showed a higher prevalence of *Staphylococcus* (70.9%) and *Pseudomonas_E_647464* (0.7%) and a lower prevalence of *Lactobacillus* (3.4%), *Bifidobacterium_388775* (3.7%), and *Escherichia_710834* (2.2%) and the GM profile at T_2_ showed a decrease in the prevalence of *Staphylococcus* (28.8%) and an increase in the prevalence of of *Pseudomonas* (4.9%) and *Escherichia_710834* (9.4%; Table 2).

**Table 2:** Faecal microbiota in case 1 at genus and species levels (showed only with percentage abundance >0.5%) were reported for T_1_ and T_2_ and gastrostomy microbiota of the upper stomach at genus and species levels (showed only with percentage abundance >0.5%) were reported for T_1_ and T_2_ (no CTRLs available).

Fecal and gastrostomy microbiota					
T_1_			T_2_		
Genus level	Patient (%)	CTRLs (%)	Genus level	Patient (%)	CTRLs (%)
**Firmicutes_D**	**79**.**9**	**26.6**	**Firmicutes_D**	**34**.**6**	**26**.**6**
*Staphylococcus*	70.9	8.1	*Staphylococcus*	28.8	8.1
*Metamycoplasma*	9.4	0.0	*Lactobacillus*	3.4	0.8
*Streptococcus*	0.5	9.8	*Faecalibacillus*	0.9	0.0
			*Streptococcus*	0.9	9.8
**Firmicutes_A**	**7**.**5**	**10.6**	**Firmicutes_A**	**17**.**3**	**28**.**6**
*Enterocloster*	1.1	0.3	*Blautia_A_141781*	6.3	0.1
*Ruminococcus_E*	1.1	0.0	*Gemmiger_A_73129*	4.7	0.2
*Gemmiger_A_73129*	0.8	0.2	*Faecalibacterium*	2.2	0.3
*Dysosmobacter*	0.7	0.0	*Agathobaculum*	1.7	0.0
*Agathobacter_164117*	0.6	0.0	*Dorea_A*	1.3	0.0
*Faecalibacterium*	1.3	0.3	*Ruminococcus_E*	1.0	0.0
**Bacteroidota**	**6**.**0**	**13.2**	**Bacteroidota**	**13**.**2**	**13**.**1**
*Phocaeicola_A_858004*	2.3	5.4	*Phocaeicola_A_858004*	4.6	5.4
*Bacteroides_H*	1.2	9.4	*Bacteroides_H*	3.3	9.4
*Alistipes_A_871400*	0.9	0.1	*Parabacteroides_B_862066*	2.2	0.2
*Barnesiella*	0.9	0.0	*Barnesiella*	1.0	0.0
**Proteobacteria**	**4**.**3**	**28.6**	*Prevotella*	1.0	0.0
*Escherichia_710834*	2.2	6.0	*Alistipes_A_871400*	0.8	0.1
*Pseudomonas_E_647464*	0.7	0.0	**Proteobacteria**	**17**.**3**	**28**.**6**
*Aeromonas*	0.6	0.0	*Pseudomonas_E_647464*	4.9	0.0
*Parasutterella*	0.6	0.0	*Burkholderia*	0.7	0.0
**Actinobacteriota**	**1**.**9**	**19.2**	*Aeromonas*	0.7	0.0
*Lawsonella*	0.9	0.0	*Klebsiella_724518*	0.0	8.2
*Cutibacterium*	0.5	0.0	*Escherichia_710834*	9.4	6.0
*Bifidobacterium_388775*	0.1	19.4	*Pseudomonas_E_647464*	4.9	0.0
**Verrucomicrobiota**	**<0.5**	**<0.5**	**Actinobacteriota**	**6**.**3**	**19**.**2**
**Firmicutes_C**	**0**.**0**	**1.6**	*Bifidobacterium_388775*	3.7	19.4
			*Corynebacterium*	1.2	0.1
			*Cutibacterium*	1.1	0.0
			**Verrucomicrobiota**	**0**.**8**	**0**.**1**
			*Akkermansia muciniphila_D_776786*	0.9	0.2
			**Firmicutes_C**	**2**.**2**	**1**.**6**
			*Veillonella_A*	1.0	1.5
Species level	Patient (%)	CTRLs (%)	Species level	Patient (%)	CTRLs (%)
**Firmicutes_D**			**Firmicutes_D**		
*Staphylococcus epidermidis_318824*	59.1	6.0	*Staphylococcus epidermidis_318824*	27.5	6.0
*Staphylococcus epidermidis_319181*	14.1	0.1	*Staphylococcus epidermidis_319181*	3.5	0.1
*Metamycoplasma hominis*	9.8	0.0	*Lactobacillus ultunensis*	3.3	0.0
*Streptococcus parasanguinis*	0.3	2.9	*Streptococcus lactarius*	0.9	0.0
**Firmicutes_A**			**Firmicutes_A**		
*Ruminococcus_E bromii*	0.9	0.0	*Blautia_A_141781 wexlerae*	2.4	0.1
*Dysosmobacter welbionis*	0.7	0.0	*Blautia_A_141781 massiliensis*	1.9	0.0
*Faecalibacterium prausnitzii_C_71358*	0.6	0.2	*Agathobacter faecis*	1.7	0.0
*Gemmiger_A_73129 qucibialis*	0.5	0.0	*Faecalibacterium prausnitzii_C_71358*	1.6	0.2
**Bacteroidota**			*Dorea_A longicatena*	1.2	0.0
*Alistipes_A_871400 putredinis*	0.9	0.0	*Gemmiger_A_73129 qucibialis*	1.1	0.0
*Barnesiella intestinihominis*	0.9	0.0	*Blautia_A_141781 faecis*	1.0	0.0
*Phocaeicola_A_858004 plebeius*	0.6	0.0	**Bacteroidota**		
*Bacteroides_H uniformis*	0.4	0.8	*Phocaeicola_A_858004 vulgatus*	3.3	5.1
*Phocaeicola_A_858004 dorei*	0.1	3.0	*Parabacteroides_B_862066 merdae*	2.4	0.1
*Phocaeicola_A_858004 vulgatus*	1.7	5.1	*Bacteroides_H uniformis*	2.0	0.8
**Proteobacteria**			*Phocaeicola_A_858004 plebeius*	1.6	0.0
*Aeromonas piscicola*	0.6	0.0	*Barnesiella intestinihominis*	1.0	0.0
**Actinobacteriota**			*Prevotella copri*	0.9	0.0
*Cutibacterium acnes*	0.5	0.0	**Proteobacteria**		
*Bifidobacterium longum*	0.1	10.9	*Burkholderia lata*	0.8	0.0
			*Aeromonas piscicola*	0.7	0.0
			**Actinobacteriota**		
			*Cutibacterium acnes*	1.1	0.0
			*Bifidobacterium pseudocatenulatum*	1.1	0.2
			*Bifidobacterium adolescentis*	1.0	0.0
			*Bifidobacterium breve*	0.4	8.2
			*Bifidobacterium bifidum*	0.0	1.1
			*Bifidobacterium longum*	1.5	10.9
**Bacteroidota**	**30**.**5**	**N.A.**	**Bacteroidota**	**3**.**2**	**N.A.**
*Phocaeicola_A_858004*	14.0	N.A.	*Phocaeicola_A_858004*	2.1	N.A.
*Bacteroides_H*	10.4	N.A.	**Firmicutes_A**	**10**.**5**	**N.A.**
*Alistipes_A_871400*	5.2	N.A.	*Faecalibacterium*	1.9	N.A.
*Prevotella*	1.7	N.A.	*CAG-217*	0.9	N.A.
*Alistipes_A_871404*	0.8	N.A.	*Lachnospira*	0.8	N.A.
**Firmicutes_A**	**29**.**8**	**N.A.**	*CAG-83*	0.7	N.A.
*Ruminococcus_E*	6.8	N.A.	*Gemmiger_A_73129*	0.6	N.A.
*Gemmiger_A_73129*	4.3	N.A.	*Ruminococcus_E*	0.6	N.A.
*Blautia_A_141781*	1.9	N.A.	*Blautia_A_141781*	0.6	N.A.
*Faecalibacterium*	1.8	N.A.	*Enterocloster*	0.5	N.A.
*Agathobacter_164117*	1.6	N.A.	**Firmicutes_D**	**74**.**3**	**N.A.**
*Copromorpha*	1.5	N.A.	*Staphylococcus*	75.7	N.A.
*Mediterraneibacter_A_155507*	1.1	N.A.	*Lactobacillus*	1.3	N.A.
*Agathobaculum*	0.8	N.A.	*Streptococcus*	0.7	N.A.
*Ruminococcus_D*	0.8	N.A.	**Proteobacteria**	**8**.**7**	**N.A.**
*Enterocloster*	0.8	N.A.	*Parasutterella*	1.7	N.A.
*Dysosmobacter*	0.8	N.A.	*Escherichia_710834*	1.1	N.A.
*Lachnospira*	0.5	N.A.	*Pseudomonas_E_647464*	1.7	N.A.
**Firmicutes_D**	**20**.**9**	**N.A.**	**Actinobacteriota**	**1**.**4**	**N.A.**
*Metamycoplasma*	11.6	N.A.	**Firmicutes_C**	**0**.**0**	**N.A.**
*Staphylococcus*	8.6	N.A.	**Verrucomicrobiota**	**1**.**0**	**N.A.**
*Faecalibacillus*	1.1	N.A.	*Akkermansia*	1.0	N.A.
**Proteobacteria**	**11**.**9**	**N.A.**	**Fusobacteriota**	**0**.**9**	**N.A.**
*Escherichia_710834*	6.3	N.A.			
*Pseudomonas_E_647464*	3.0	N.A.			
*Aeromonas*	1.0	N.A.			
*Parasutterella*	0.5	N.A.			
**Actinobacteriota**	**3**.**1**	**N.A.**			
*Cutibacterium*	1.9	N.A.			
*Bifidobacterium_388775*	0.7	N.A.			
**Firmicutes_C**	**2**.**7**	**N.A.**			
*Veillonella_A*	1.2	N.A.			
*Dialister*	0.6	N.A.			
**Verrucomicrobiota**	**1**.**0**	**N.A.**			
*Akkermansia*	1.1	N.A.			

**Species level**	**Patient (%)**	**CTRLs (%)**	**Species level**	**Patient (%)**	**CTRLs (%)**
**Bacteroidota**			**Bacteroidota**		
*Phocaeicola_A_858004 vulgatus*	10.3	N.A.	*Phocaeicola_A_858004 vulgatus*	0.6	N.A.
*Bacteroides_H uniformis*	3.3	N.A.	*Phocaeicola_A_858004 dorei*	1.4	N.A.
*Phocaeicola_A_858004 dorei*	2.3	N.A.	**Firmicutes_A**		
*Prevotella copri*	1.8	N.A.	*CAG-217 sp000436335*	1.0	N.A.
*Bacteroides_H fragilis*	1.7	N.A.	*Lachnospira eligens*	0.8	N.A.
*Phocaeicola_A_858004 plebeius*	1.7	N.A.	*CAG-83 sp900545585*	0.7	N.A.
*Alistipes_A_871400 shahii*	1.4	N.A.	*Gemmiger_A_73129 qucibialis*	0.5	N.A.
*Bacteroides_H caccae*	1.2	N.A.	*Faecalibacterium prausnitzii_C_71358*	1.7	N.A.
*Bacteroides_H eggerthii*	1.0	N.A.	**Firmicutes_D**		
**Firmicutes_A**			*Staphylococcus epidermidis_319181*	10.3	N.A.
*Gemmiger_A_73129 qucibialis*	3.7	N.A.	*Lactobacillus ultunensis*	1.1	N.A.
*Ruminococcus_E bromii*	3.6	N.A.	*Streptococcus lactarius*	0.5	N.A.
*Ruminococcus_E bromii_B*	2.3	N.A.	*Staphylococcus epidermidis_318824*	66.6	N.A.
*Other species*	2.0	N.A.	**Proteobacteria**		
*Copromorpha sp900066305*	1.6	N.A.	*Parasutterella gallistercoris*	1.7	N.A.
*Faecalibacterium prausnitzii_C_71358*	1.5	N.A.	**Verrucomicrobiota**		
*Agathobacter rectalis*	1.4	N.A.	*Akkermansia muciniphila_D_776786*	1.0	N.A.
*Ruminococcus_E sp003438075*	1.4	N.A.			
**Firmicutes_D**					
*Metamycoplasma hominis*	12.5	N.A.			
*Staphylococcus epidermidis_318824*	7.0	N.A.			
*Staphylococcus epidermidis_319181*	1.2	N.A.			
*Faecalibacillus intestinalis*	1.1	N.A.			
**Proteobacteria**					
*Aeromonas piscicola*	1.1	N.A.			
*Parasutterella gallistercoris*	0.6	N.A.			
**Actinobacteriota**					
*Cutibacterium acnes*	2.0	N.A.			
**Firmicutes_C**					
*Veillonella_A rogosae*	1.3	N.A.			
*Dialister invisus*	0.6	N.A.			
**Verrucomicrobiota**					
*Akkermansia muciniphila_D_776786*	1.2	N.A.			

Bold values indicate phylum-level identification, whereas non-bold values indicate genus-level identification.

The comparison of gastric stoma content of the upper portion at T_1_ and T_2_ was also evaluated, showing both a high prevalence of Firmicutes (especially Firmicutes_A and Firmicutes_D) and Proteobacteria and a low prevalence of Actinobacteriota at T_1_ and at T_2_. In particular, at T_2_, the gastric profile showed an increase in the prevalence of Firmicutes (74.3% of Firmicutes_D and 10.5% of Firmicutes_A) and a decrease in the prevalence of Bacteroidota (3.2%) and Actinobacteriota (1.4%) compared with that at T_1_ profile (Figures 2a,b). Although the gastrostomy content at T_1_ showed a lower prevalence of *Staphylococcus* (8.6%) and a higher prevalence of *Phocaeicola_A_858004* (14%) *and Bacteroides_H* (10.4%) compared with that at T_2_, the latter time point was characterized by an increase in the prevalence of *Staphylococcus* (75.7%) and a decrease in the prevalence of *Phocaeicola_A_858004* (2.1%; Table 2).

Finally, analysis of LPS on serum samples was performed at T_1_ and T_2_, and its results showed that higher lipopolysaccharide levels corresponded to the higher prevalence of proteobacteria at T_2_ (7.32 EU/mL) compared with those at T_1_ (4.99 EU/mL).

### Case 2

#### Clinical presentation

A preterm male neonate (GA: 24 + 6 weeks) was transferred to our center for the management of a large patent ductus arteriosus (PDA) and had a prior ileostomy due to a previous NEC. Because of ulceration of ileostomy, perforation, and fibrous scar tissue formation between the gut and the peritoneum with ileal strictures, the patient underwent a further ileoileal resection and anastomosis with the removal of the perforated area, including the terminal ileum, ileocecal valve, cecum, ascending colon, and hepatic flexure. An ileocolostomy with a counteropening in the controlateral flank was performed. Consequently, the infant underwent transcatheter PDA closure with a Piccolo Occluder device. A transit X-ray scan at 2 months showed good results, and the patient tolerated well enteral feeding initially with fortified donor human milk and was then shifted to hydrolyzed formula before discharge.

During the hospital stay, the patient was treated for *Klebsiella pneumoniae* sepsis with a regimen of meropenem and intravenous immunoglobulins. He also received vancomycin and amikacin for a culture-negative late-onset sepsis.

#### Gut microbiota

The GM profile showed a prevalence of Firmicutes (77.5% of Firmicutes_D and 18.7% of Firmicutes_A) and Proteobacteria (3.5%). In particular, an increase in the *Staphylococcus* (70.9%) and *Proteus* (2.3%) populations and a decrease in *Bifidobacterium_388775* (<0.1%) and *Akkermansia muciniphila_D_776786* (<0.1%) populations were observed in this patient compared with corresponding microbial populations in age-matched CTRLs. The profile was also characterized by a decrease in Bacteroidota (0.2%) and Actinobacteriota (<0.1%) populations (Figure 3, Table 3). Moreover, the serum sample collection was performed simultaneously with that of the gut microbiota, and serum LPS analysis showed high LPS levels (5.1 EU/mL).

**Figure 3:**
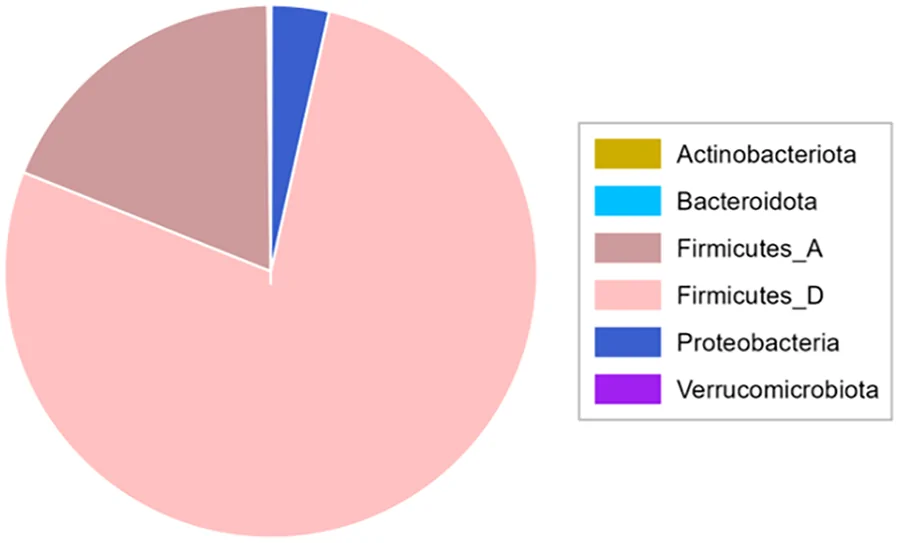
The GM profile of “case 2” on a stool sample after surgery based on a comparison with CTRLs. The GM profile of “case 2” on a single stool sample, a second-level report, based on a comparison with CTRLs.

**Table 3:** Fecal microbiota at genus and species levels in case 2 (showed only with percentage abundance >0.5%).

Genus level	Patient (%)	CTRLs (%)	Species level	Patient (%)	CTRLs (%)
**Firmicutes_D**	**77**.**5**	**22**.**6**	**Firmicutes_D**		
*Staphylococcus*	70.9	0.2	*Enterococcus_H_360604 faecalis*	4.1	3.9
*Enterococcus_H_360604*	3.9	3.7	*Streptococcus oralis_E_351036*	0.2	1.4
*Enterococcus_B*	2.4	0.1	*Streptococcus lactarius*	0.2	1.2
*Streptococcus*	0.5	8.9			
**Firmicutes_A**	**18**.**7**	**15**.**4**	**Firmicutes_A**		
*Clostridioides_A*	18.6	0.6	*Mediterraneibacter_A_155507 torques*	0.0	0.6
*Mediterraneibacter_A_155507*	0.0	0.6	*Clostridioides_A difficile*	19.8	0.3
**Proteobacteria**	**3**.**5**	**12**.**3**	**Proteobacteria**		
*Proteus*	2.3	0.0	*Klebsiella pneumoniae_718977*	0.0	0.6
*Escherichia_710834*	1.0	6.1	*Proteus mirabilis*	2.4	0.0
*Klebsiella_724518*	0.2	2.8			
**Bacteroidota**	**0**.**2**	**10**.**3**	**Bacteroidota**		
*Phocaeicola_A_858004*	0.1	3.2	*Phocaeicola_A_858004 vulgatus*	0.1	3.3
*Bacteroides_H*	0.1	5.8	*Bacteroides_H uniformis*	0.0	3.5
			*Bacteroides_H xylanisolvens*	0.0	1.7
**Actinobacteriota**	**0**.**0**	**33**.**2**	**Actinobacteriota**		
*Bifidobacterium_388775*	0.0	32.8	*Bifidobacterium adolescentis*	0.0	0.5
*Corynebacterium*	0.0	1.4			
**Verrucomicrobiota**	**0**.**0**	**0**.**3**	**Verrucomicrobiota**	<0.5	
*Akkermansia muciniphila_D_776786*	<0.5	N.A.			

Bold values indicate phylum-level identification, whereas non-bold values indicate genus-level identification.

### Case 3

#### Clinical presentation

A male newborn was born at 38 weeks of GA via a planned cesarean section at term due to breech presentation and had a birthweight of 2,750 g. The immediate postnatal adaptation was satisfactory, with Apgar scores of 8 and 9. On physical examination, features suggestive of Down syndrome were noted, and karyotype analysis confirmed the diagnosis of trisomy 21.

Shortly after birth, the infant developed signs of neonatal intestinal obstruction, including failure to pass meconium, progressive abdominal distension, and bilious vomiting. The patient was then transferred to a tertiary care center for further evaluation and management. Cardiorespiratory function remained stable throughout hospitalization. The infant was consistently eupneic in room air and maintained good hemodynamic parameters. Early echocardiographic evaluation revealed a small atrial septal defect, a small patent ductus arteriosus, and a right retroesophageal subclavian artery. These findings were not hemodynamically significant but were scheduled for follow-up.

Upon admission, the intestinal obstruction was initially managed conservatively with frequent rectal irrigations, which provided partial decompression and allowed the initiation of enteral nutrition. Both congenital hypothyroidism and cystic fibrosis were ruled out. A rectal suction biopsy was performed, showing fragments of colonic mucosa and submucosa with preserved crypt architecture. Submucosal nerve trunks were present, but ganglion cells were absent. Histoenzymatic staining revealed increased acetylcholinesterase (ACE)–positive nerve fibers in the lamina propria, while immunohistochemistry for calretinin confirmed the absence of intrinsic innervation. These findings supported the diagnosis of aganglionic megacolon.

The patient continued to respond well to rectal irrigations throughout hospitalization. The technique was taught to the caregivers, who were able to perform it effectively, ensuring bowel decompression and symptom control. There were no infectious or metabolic complications during the hospital stay. Feeding with hydrolyzed formula was well tolerated, and the infant demonstrated regular weight gain and stable clinical status. Over time, the stool output became less voluminous and more formed, allowing for gradual weight gain and improved metabolic balance.

Renal and urinary function remained normal throughout the hospital stay. Diuresis was appropriate, and renal ultrasound results showed no structural abnormalities. Neurologically, no overt deficits were observed. A mild, non-specific periventricular echogenicity was noted on a cranial ultrasound, but it was not associated with clinical concerns. No infectious complications occurred during the admission. Hematologically, the infant experienced transient thrombocytopenia during the early neonatal period, which resolved spontaneously, and chronic anemia that required red blood cell transfusion and iron and vitamin supplementation. At discharge, the hemoglobin levels were within acceptable limits.

Routine vaccinations were administered and well tolerated. The patient was discharged at approximately 4 months of age in stable clinical condition with plans for a multidisciplinary follow-up, surgical re-evaluation, and nutritional support.

#### Gut microbiota

The GM composition showed an overabundance of Firmicutes (in particular, 59.6% of Firmicutes_D) and Proteobacteriota (39.3%) compared with that of age-matched CTRLs; in particular, an increase in *Streptococcus* (48.8%) and *Lactobacillus* (7.2%) populations but a decrease in *Veillonella_A* (<1%) and *Faecalibacterium* (<0.1%) populations was observed. Moreover, an increase in *Escherichia_710834* (39.2%) abundance was reported compared with that in CTRLs. The GM profile was also characterized by a decrease in Bacteroidota and Actinobacteriota populations, especially *Phocaeicola_A_858004* (<0.1%), *Bacteroides_H* (<0.1%), and *Bifidobacterium_388775* (0.8%), compared with that of CTRLs (Figure 4A; Table 4). The GM profile on three stool samples showed a high dysbiosis index (36%; Figure 4B).

**Figure 4:**
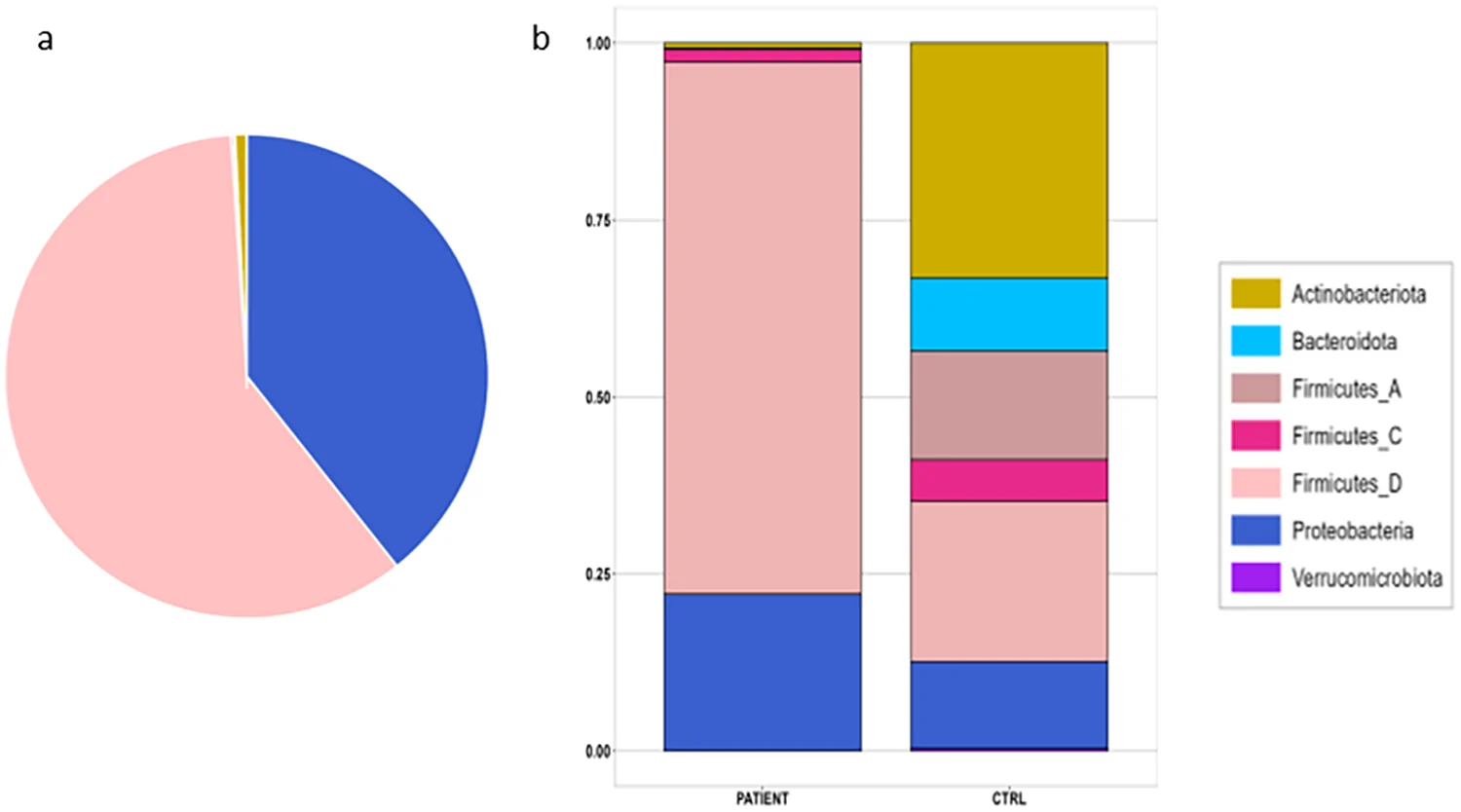
The GM profile of “case 3” on a single stool sample **(a)**, a second-level report, and on three stool samples **(b)**, a dysbiosis-based report based on a comparison with CTRLs.

**Table 4:** Fecal microbiota at the genus and species levels in case 3 (showed only with percentage abundance >0.5%).

Genus level	Patient (%)	CTRLs (%)	Species level	Patient (%)	CTRLs (%)
**Firmicutes_D**	**59**.**6**	**22**.**6**	**Firmicutes_D**		
*Streptococcus*	48.8	8.9	*Lactobacillus gasseri_329736*	7.1	1.1
*Lactobacillus*	7.2	1.1	*Limosilactobacillus reuteri*	1.7	0.0
*Limosilactobacillus*	3.6	0.0	*Streptococcus oralis_E_351036*	0.0	1.4
*Gemella*	0.0	0.6	*Enterococcus_H_360604 faecalis*	0.0	3.9
*Enterococcus_H_360604*	0.0	3.7			
**Proteobacteria**	**39**.**3**	**12**.**3**	**Proteobacteria**		
*Escherichia_710834*	39.2	6.1	*Haemophilus_D_735815 parainfluenzae*	0.0	1.6
*Haemophilus_D_735815*	0.0	2.3			
**Actinobacteriota**	**0**.**8**	**33**.**2**	**Actinobacteriota**		
*Bifidobacterium_388775*	0.8	32.8	*Bifidobacterium animalis*	0.7	0.0
			*Bifidobacterium breve*	0.1	22.3
			*Bifidobacterium longum*	0.0	8.3
**Firmicutes_A**	**0**.**2**	**15**.**4**	**Firmicutes_A**		
*Faecalibacterium*	0.0	1.1	*Mediterraneibacter gnavus*	0.0	2.0
*Mediterraneibacter_A_155507*	0.0	0.6			
*Ruminococcus_B*	0.0	1.9			
**Bacteroidota**	**0**.**0**	**10**.**3**	**Bacteroidota**		
*Phocaeicola_A_858004*	0.0	3.2	*Bacteroides_H uniformis*	0.0	3.5
*Bacteroides_H*	0.0	5.8	*Phocaeicola_A_858004 vulgatus*	0.0	3.3
**Firmicutes_C**	**0**.**0**	**5**.**8**	**Firmicutes_C**		
*Veillonella_A*	0.0	6.4			
**Verrucomicrobiota**	**0**.**0**	**0**.**3**			

Bold values indicate phylum-level identification, whereas non-bold values indicate genus-level identification.

### Case 4

#### Clinical presentation

A female neonate born at 39 weeks of GA with a birthweight of 3,430 g was admitted to our center at five days of life due to the absence of meconium passage and progressive abdominal distension. Antibiotic treatment with ampicillin, netilmicin, and metronidazole was started empirically from the transferring hospital with the suspicion of a gut perforation. Clinical and radiological findings were consistent with intestinal obstruction. A contrast enema was performed, which raised suspicion for megacolon and Hirschsprung’s disease. This was followed by a rectal suction biopsy, which confirmed the diagnosis. Microscopic examination of the biopsy revealed fragments of colonic mucosa and submucosa with preserved crypt architecture and mucin production. Submucosal nerve trunks were present without ganglion cells (peripherin-negative). ACE histoenzymatic staining showed an increase in proliferation of cholinergic nerve fibers within the lamina propria, and immunohistochemistry with anticalretinin confirmed the absence of intrinsic nerve fibers, supporting the diagnosis of aganglionosis. Despite the aganglionic segment, the patient responded well to conservative management with rectal irrigations, which were continued throughout the hospital stay. The nursing technique was taught to and successfully adopted by the caregivers, allowing effective decompression of the bowel without immediate need for surgical intervention.

At discharge, the infant was in good general condition, tolerating full enteral feeds, with normal weight gain and stable clinical parameters. Rectal irrigations remained effective in managing bowel function. Plans were made for outpatient follow-up and reassessment to determine the appropriate timing for definitive future surgical treatment.

#### Gut microbiota

The GM profile showed an overabundance of Firmicutes (99.1% of Firmicutes_D) compared with the GM profile of age-matched CTRLs, which showed high levels of *Streptococcus* (99.1%). The profile was also characterized by a deprivation of Bacteroidetes and Actinobacteria, especially a decrease in *Bacteroides_H* (< 0.1%) and *Bifidobacterium_388775* (< 0.1%) populations (Figure 5A; Table 5). The GM profile on three stool samples showed a high level of dysbiosis (42%; Figure 5B).

**Figure 5:**
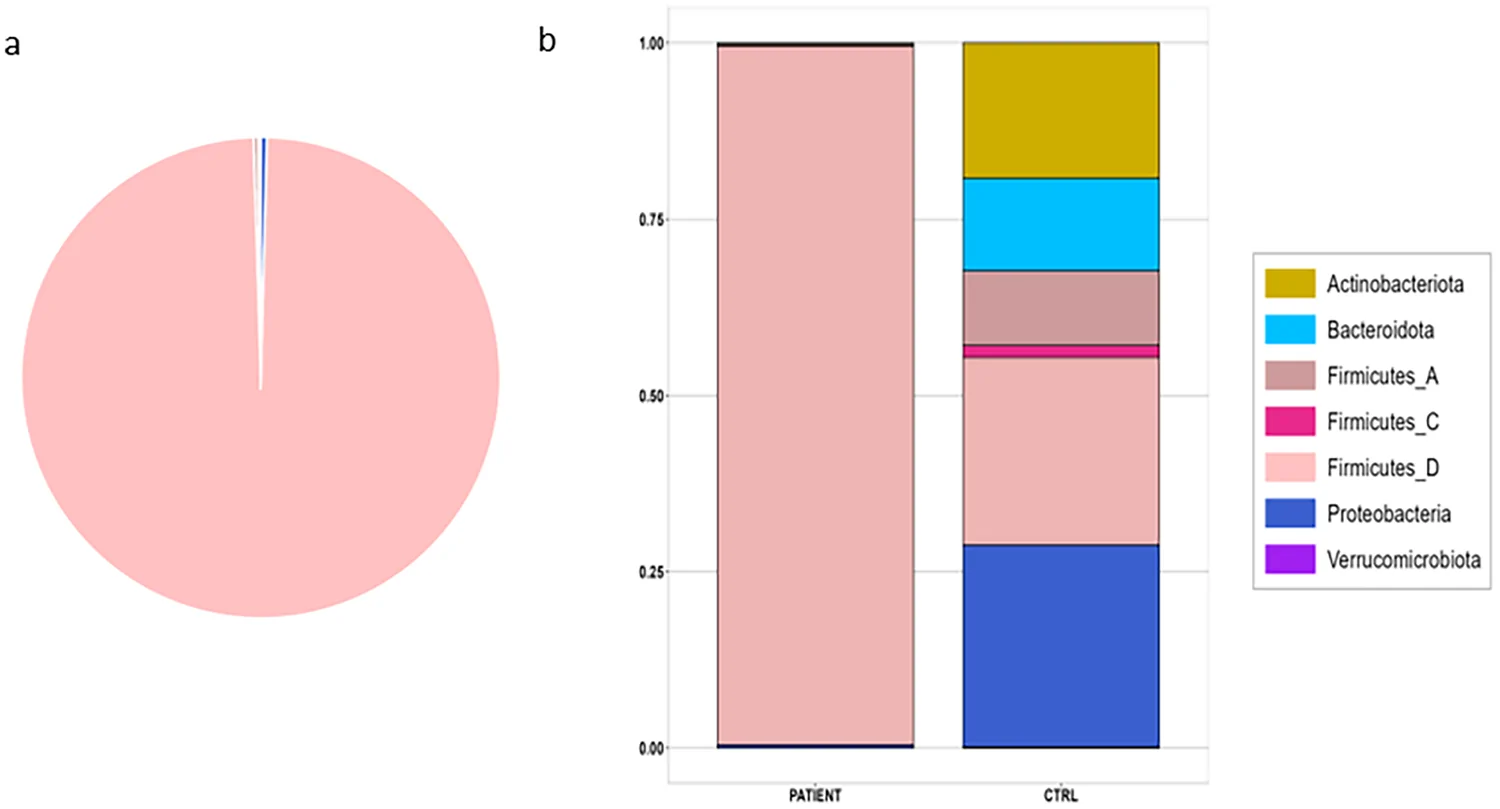
The GM profile of “case 4” performed on a single stool sample **(a)**, a second-level report, and on three stool samples **(b)**, a dysbiosis-based report based on a comparison with CTRLs.

**Table 5:** Fecal microbiota at the genus and species levels in case 4 (showed only with percentage abundance >0.5%).

Genus level	Patient (%)	CTRLs (%)	Species level	Patient (%)	CTRLs (%)
**Firmicutes_D**	**99**.**1**	**26**.**6**	**Firmicutes_D**		
*Streptococcus*	99.1	9.8	*Streptococcus oralis_E_351036*	0.5	0.4
*Lactobacillus*	0.0	0.8	*Staphylococcus epidermidis_318824*	0.0	6.0
*Staphylococcus*	0.0	8.1	*Streptococcus lactarius*	98.5	0.0
**Proteobacteria**	**0**.**4**	**28**.**6**	**Proteobacteria**		
*Klebsiella_724518*	0.4	8.2	*Klebsiella variicola*	0.1	0.8
*Escherichia_710834*	0.0	6.0	*Klebsiella pneumoniae_718977*	0.1	1.6
**Firmicutes_A**	**0**.**3**	**10**.**6**			
**Bacteroidota**	**0**.**1**	**13**.**2**	**Bacteroidota**		
*Phocaeicola_A_858004*	0.1	5.4	*Phocaeicola_A_858004 dorei*	0.0	3.0
*Bacteroides_H*	0.0	9.4	*Bacteroides_H uniformis*	0.0	0.8
			*Phocaeicola_A_858004 vulgatus*	0.0	5.1
**Actinobacteriota**	**0**.**1**	**19**.**2**	**Actinobacteriota**		
*Bifidobacterium_388775*	0.0	19.4	*Rothia sp001808955*	0.0	0.5
			*Bifidobacterium bifidum*	0.0	1.1
			*Bifidobacterium longum*	0.0	10.9

Bold values indicate phylum-level identification, whereas non-bold values indicate genus-level identification.

### Case 5

#### Clinical presentation

A male term neonate, born at 38 weeks of GA with a birthweight of 3,280 g, was admitted to the neonatal surgical ward for evaluation and management of suspected posterior urethral valves (PUVs). Initial clinical presentation was marked by signs of urinary tract infection and poor urinary flow. A bladder catheter was positioned, and empirical antibiotic therapy was started with ampicillin and netilmicin. Urine and blood cultures yielded *S. aureus*, confirming both urinary and hematogenous spreading. Targeted intravenous antibiotic treatment with teicoplanin was provided, leading to clinical and microbiological resolution of the infection.

In the following days, the clinical course was complicated by a second episode of sepsis caused by *K. pneumoniae*, which was treated successfully with intravenous meropenem. During the early phase of hospitalization, the infant experienced transient polyuria, requiring intravenous fluid and electrolyte support to maintain homeostasis.

A voiding cystourethrogram confirmed the presence of posterior urethral valves and revealed severe bilateral vesicoureteral reflux, classified as grade V. The patient underwent multidisciplinary evaluation that involved the nephrology and urology teams. Bladder catheterization was maintained throughout the diagnostic workup and treatment period.

At the time of discharge (1 month of age), the infant was in good general condition and feeding well by mouth. The bladder catheter was in place and functioning appropriately, with no signs of obstruction or leakage. Follow-up plans included surgical correction of the PUVs and monitoring for potential renal sequelae.

#### Gut microbiota

The GM profile showed an increase in the abundance of Firmicutes (especially 40.7% of Firmicutes_A and 31.4% of Firmicutes_D) and of Proteobacteriota (19.9%) and a decrease in the abundance of Bacteroidota (6.7%), Actinobacteriota (1.3%), and Verrucomicrobiota (<0.1%) compared with the GM profile of age-matched CTRLs. In particular, proteobacteriota were characterized by increases in *Klebsiella_724518* (13.5%), *Proteus* (7%), *Escherichia_710834* (2.3%), and *Pseudomonas_E_647464* (0.9%) populations (Figure 6A; Table 6). The GM profile on three stool samples showed a high level of dysbiosis (37%; Figure 6B).

**Figure 6:**
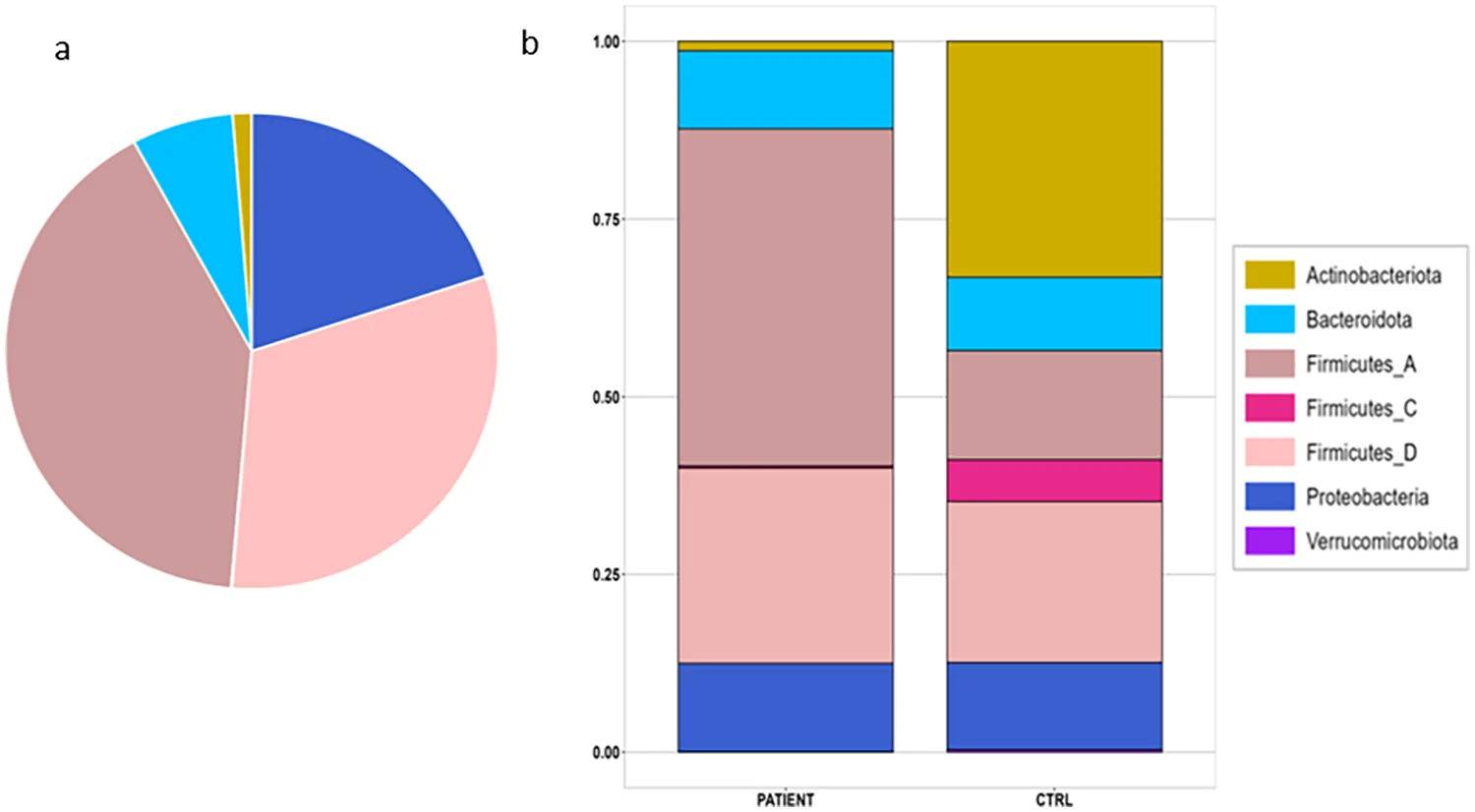
The GM profile of “case 5” performed on a single stool sample **(a)**, a second-level report, and on three stool samples **(b)**, a dysbiosis-based report based on a comparison with CTRLs.

**Table 6:** Fecal microbiota at the genus and species levels in case 5 (showed only with percentage abundance >0.5%).

Genus level	Patient (%)	CTRLs (%)	Species level	Patient (%)	CTRLs (%)
**Firmicutes_A**	**40**.**7**	**15**.**4**	**Firmicutes_A**		
*Clostridioides_A*	7.0	0.6	*Dorea_A longicatena*	1.3	0.1
*Gemmiger_A_73129*	3.0	0.5	*Acetatifactor sp900066565*	1.2	0.1
*Dorea_A*	1.7	0.1	*Fimenecus sp000432435*	1.2	0.1
*Agathobacter_164117*	1.3	0.1	*Agathobacter rectalis*	0.9	0.1
*Acetatifactor*	1.2	0.1	*Faecalibacterium prausnitzii_C_71358*	0.9	0.5
*Fimenecus*	1.2	0.1	*Limivicinus sp002320035*	0.9	0.0
*Faecalibacterium*	1.1	1.1	*Mediterraneibacter_A_155507 torques*	0.8	0.6
*Limivicinus*	0.9	0.0	*ER4 sp900317525*	0.8	0.0
*Mediterraneibacter_A_155507*	0.8	0.6	*Fusicatenibacter saccharivorans*	0.7	0.0
*Blautia_A_141781*	0.8	0.3	*Clostridioides_A difficile*	7.1	0.3
**Firmicutes_D**	**31**.**4**	**22**.**6**	**Firmicutes_D**		
*Enterococcus_B*	20.5	0.1	*Streptococcus lactarius*	6.6	1.2
*Streptococcus*	6.9	8.9	*Lactobacillus delbrueckii*	5.4	0.0
*Lactobacillus*	5.3	1.1	*Lactococcus_A_346120 lactis_344179*	3.2	0.0
*Lactococcus_A_346120*	3.1	0.0	*Enterococcus_H_360604 faecalis*	0.6	3.9
*Enterococcus_H_360604*	0.6	3.7	*Streptococcus oralis_E_351036*	0.5	1.4
**Proteobacteria**	**19**.**9**	**12**.**3**	**Proteobacteria**		
*Proteus*	7.0	0.0	*Klebsiella pneumoniae_718977*	7.0	0.6
*Escherichia_710834*	2.3	6.1	*Klebsiella variicola*	5.1	0.5
*Pseudomonas_E_647464*	0.9	0.0	*Proteus mirabilis*	7.1	0.0
*Klebsiella_724518*	13.5	2.8			
**Bacteroidota**	**6**.**7**	**10**.**3**	**Bacteroidota**		
*Phocaeicola_A_858004*	3.0	3.2	*Phocaeicola_A_858004 dorei*	1.1	0.0
*Bacteroides_H*	1.8	5.8	*Bacteroides_H uniformis*	1.0	3.5
*Parabacteroides_B_862066*	1.2	0.4	*Parabacteroides_B_862066 merdae*	0.9	0.4
*Prevotella*	1.0	0.1	*Barnesiella intestinihominis*	0.8	0.0
*Barnesiella*	0.8	0.0	*Prevotella rara*	0.6	0.0
*Alistipes_A_871400*	0.1	0.6	*Bacteroides_H xylanisolvens*	0.1	1.7
			*Phocaeicola_A_858004 vulgatus*	1.6	3.3
			*Phocaeicola_A_858004 dorei*	1.1	0.0
**Actinobacteriota**	**1**.**3**	**33**.**2**	**Actinobacteriota**		
*Bifidobacterium_388775*	0.4	32.8	*Bifidobacterium longum*	0.2	8.3
*Corynebacterium*	0.2	1.4			
**Firmicutes_C**	**0**.**1**	**5**.**8**	**Firmicutes_C**		

Bold values indicate phylum-level identification, whereas non-bold values indicate genus-level identification.

## Discussion

This study is, to our knowledge, one of the few studies to explore the gut microbiota alterations of newborns in relation to different complex diseases including surgical, gastrointestinal, and urological conditions and to clinical factors potentially influencing the microbial composition, such as antibiotic exposure, prematurity, and feeding modality. With this aim, the GM analysis of five newborns has allowed us to highlight potential signatures of dysbiosis and disease-related microbial patterns. In particular, we have observed a consistent reduction in the population of promising beneficial commensals, such as *Bifidobacterium* and *Lactobacillus*, and a parallel overgrowth of potential opportunistic pathogens, mainly within the phylum Proteobacteriota. These findings support the notion that different factors in early life may affect microbial colonization and contribute to complications and systemic inflammation.

The study by Rao et al. reported that the altered microbiota environment after a gastrointestinal surgery can favor the growth of potential opportunistic pathogens such as *Staphylococcus* and species belonging to the *Enterobacteriaceae* family (such as *Escherichia* and *Klebsiella*). Moreover, surgery may be associated with alterations in the gut microbial community, including a reduction in potentially beneficial taxa such as *Bifidobacterium* and changes in the relative abundance of taxa including *Pseudomonas* and *Escherichia* (32). However, these microbial shifts should not be considered only as specific signatures of surgery or disease, as they may be influenced by multiple clinical and environmental factors.

Gastrointestinal surgery in newborns and clinical factors, such as antibiotic exposure, preterm birth, and feeding, can significantly disrupt the GM, possibly leading to intestinal dysbiosis, which is characterized by reduced complexity and an overgrowth of potential opportunistic pathogens. Indeed, these infants usually exhibit a decrease in the variety and abundance of different bacterial species with a less degree of diversity (15). In particular, gastrostomy can influence the colonization of the stomach in neonates. Gastrostomy content cultures from infants, despite culture-based microbiological assessment and microbiota profiling methodologies not being directly comparable, were characterized by an increase in Proteobacteriota (especially Enterobacteriaceae, such as *Escherichia* and *Klebsiella*, and *Pseudomonas* spp.) and Firmicutes, with particular reference to *Staphylococcus* (i.e., *Staphylococcus epidermidis*) successfully providing isolated colonies, which were also isolated by cultures. There are several possible explanations for this abnormal gut microbiota profile compared with the gastric content of premature neonates without gastrostomy, including the growth of bacteria on the catheter and the reflux of gastric contents between the catheter and the stomach (33). Moreover, overgrowth of Firmicutes, especially *Staphylococcus* (i.e., *Staphylococcus epidermidis*), in the GM may reside in the preterm birth, since this condition is usually associated with a higher abundance of Proteobacteriota and coagulase-negative Staphylococci (CoNS; such as *S. epidermidis*) than in the GM of at-term neonates. Indeed, *S. epidermidis* can cause infections especially in patients with weakened immune systems, such as preterm infants or infants with implanted medical devices (34). Moreover, lower abundances of promising beneficial species, such as *Lactobacillus*, *Ruminococcus*, and *Bifidobacterium*, are associated with immunological impairment in preterm neonates (14, 19–21). Interestingly, “case 1” presented positive results on BAL and bronchial aspirate cultures for *S. maltophilia* (Proteobacteria), which often can be associated with iatrogenic multidrug resistant (MDR) colonization (35).

Microbial diversity in neonatal ileostomy content was observed as generally low, was dominated by members of the phyla Proteobacteriota (i.e., *Pseudomonas* and *Escherichia* spp.) and Firmicutes, and had a low abundance of Bacteroidota, suggesting an unhealthy state of the small intestine microbiota after ileostomy (36). Hence, a study on the patient with resection reported a reduction in the Bacteroidetes population and an overabundance of Firmicutes compared with the GM profile of their CTRLs, leading to an increased Firmicutes/Bacteroidetes ratio after small bowel resection (37).

Therefore, for “case 1” and “case 2,” intestinal dysbiosis can be identified as a possible “signature” in neonatal patients undergoing surgery. In particular, in these patients, dysbiosis was associated with deficiency of *Bacteroides* and *Bifidobacterium* populations and abundance of *Escherichia* and *Pseudomonas*—in general, abundance of Proteobacteria. Another characteristic of both cases are the high levels of serum LPS that can be correlated with the abundance of Gram-negative bacteria such as Proteobacteria, and the possible correlated peripheral inflammation

With regard to Hirschsprung's disease, one of the main congenital gastrointestinal conditions that require surgery in the neonatal period, intestinal dysbiosis has been reported, including a reduction in the population of potentially beneficial taxa such as *Bacteroides* and *Bifidobacterium* and an overabundance of *Escherichia*. Moreover, the microbiota of patients with Hirschsprung-associated enterocolitis (HAEC) and those in HAEC remission (HAEC-R) has been characterized by an overabundance of Proteobacteriota and Firmicutes and an overreduction of Bacteroidota (38). Specifically, for “case 3” and “case 4,” dysbiosis signatures, identified as characteristics of Hirschsprung’s disease, were observed, which were particularly associated with the overabundance of Proteobacteriota (as observed in “case 3”) and Firmicutes (as observed in “case 4”), while an overreduction of Bacteroidota and Actinobacteriota populations with a reduction in abundance of promising eubiotic bacteria such as Bacteroides and Bifidobacterium in both cases were observed. For “case 3,” additional signatures (i.e., an overabundance of Proteobacteriota such as *Escherichia* spp.) were observed. However, these microbial shifts may be influenced by multiple clinical and environmental factors.

In “case 5,” GM alterations in neonates with complex urological conditions, such as a decrease in abundance of short-chain fatty acid (SCFA)–producing bacteria (such as *Bifidobacterium*) and an increase in abundance of potential pathogenic bacteria such as *Enterobacteriaceae* (*Klebsiella* and *Escherichia*), were observed (39). Specifically, their GM is dominated by aerobic taxa, along with an increase in the population of aerobic microorganisms, such as *Klebsiella* spp., *Proteus vulgaris*, and *P. aeruginosa*, and a decrease in anaerobic taxa (40).

Nevertheless, our study has several limitations that should be acknowledged. First, the small sample size (five neonates) does not allow for generalizable conclusions and may not capture the full variability of GM alterations in this clinical setting. Second, the heterogeneity of surgical conditions (gastrostomy, ileostomy, Hirschsprung’s disease, and bowel resection) and baseline characteristics of patients (preterm vs. term birth, antibiotic exposure, and nutritional regimens) may have influenced microbial profiles independently of surgery. Third, there is difficulty in distinguishing surgery-specific microbial changes from those related to prematurity, hospitalization, underlying disease, or other clinical factors. In addition, the timing of sample collection was not completely uniform among patients, which may have introduced variability related to postoperative time and clinical status.

## Conclusions

To conclude, this case series highlights how the GM of neonates with complex surgical, gastrointestinal, and urological conditions is associated with significant intestinal dysbiosis, marked by loss of beneficial commensals and overgrowth of potential opportunistic pathogens. Despite the limited sample size, consistent microbial signatures emerged across different pathological conditions and in relation to various factors in early life, including antibiotic therapy, premature birth, and feeding. These findings underline the importance of monitoring the microbiota composition in neonates with complex gastrointestinal and urological conditions, including surgery, and the need for larger, longitudinal studies to evaluate the long-term impact of dysbiosis and the potential role of microbiota-targeted interventions in improving outcomes.

## Data Availability

Publicly available datasets were analyzed in this study. This data can be found here: https://www.ncbi.nlm.nih.gov/bioproject/?term=PRJNA1517820.
